# Successful implantation of a left ventricular lead for cardiac resynchronization therapy upgrade via an occluded subclavian vein using the balloon-target puncture technique

**DOI:** 10.1093/ehjcr/ytae110

**Published:** 2024-02-23

**Authors:** Yuhei Kasai, Junji Morita, Takuya Haraguchi, Takayuki Kitai

**Affiliations:** Department of Cardiology, Sapporo Cardiovascular Clinic, North 49, East 16, 8-1, Higashi Ward, Sapporo, Hokkaido 007-0849, Japan; Department of Cardiology, Sapporo Cardiovascular Clinic, North 49, East 16, 8-1, Higashi Ward, Sapporo, Hokkaido 007-0849, Japan; Department of Cardiology, Sapporo Cardiovascular Clinic, North 49, East 16, 8-1, Higashi Ward, Sapporo, Hokkaido 007-0849, Japan; Department of Cardiology, Sapporo Cardiovascular Clinic, North 49, East 16, 8-1, Higashi Ward, Sapporo, Hokkaido 007-0849, Japan

## Case description

**Figure 1:**
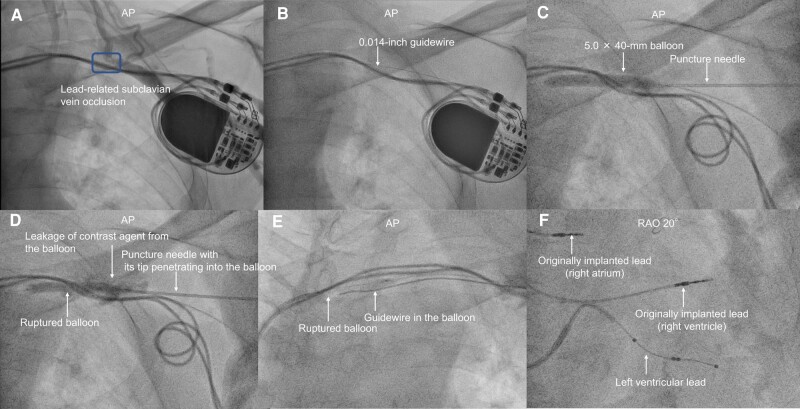
(*A*) Contrast venography via the left brachial vein. (*B*) A 0.014-inch guidewire successfully traversed the subclavian vein occlusion. (*C*, *D*) The puncture needle pierced the inflated balloon, ensuring that it did not overlap with any other leads under fluoroscopic guidance. (*E*) After advancing the guidewire and the ruptured balloon simultaneously, we then pushed the balloon further while guiding the wire from the balloon. (*F*) Successful implantation of a left ventricular lead in the posterolateral branch.

An 84-year-old man was referred for upgrading a permanent pacemaker (complete atrioventricular block) to cardiac resynchronization therapy (CRT) because of symptomatic heart failure with reduced left ventricular ejection fraction (26%) from pacemaker-induced cardiomyopathy (ventricular pacing rate: 100%) without a structural heart disease. Contrast venography via the left brachial vein showed that lead-related subclavian vein (SCV) occlusion had occurred (*Figure 1A*). The occlusion length was short, leading to the decision to perform endovascular treatment. A 6Fr Glidesheath (Terumo, Tokyo, Japan) was inserted into the left brachial vein. A 0.014-inch guidewire (Gladius; Asahi Intecc, Aichi, Japan) with a microcatheter (Corsair PV; Asahi Intecc) was successfully passed through the occlusion (*Figure 1B*). Balloon angioplasty was performed using a 5.0 × 40-mm balloon (JADE; OrbusNeich, Hong Kong), followed by an attempt to puncture the left SCV from its pocket. This attempt was unsuccessful owing to venous elastic recoil. Subsequently, we punctured the inflated balloon, ensuring it did not overlap with any other leads (*Figure 1C* and *D*). We then inserted a guidewire into the balloon and advanced both the balloon and guidewire. Thereafter, we pushed only the balloon forward, positioning the guidewire outside the balloon, thereby securing an access route for the left ventricular lead (*Figure 1E*, Supplementary material online, *Video S1*). This series of steps is called the balloon-target puncture technique. We successfully performed implantation of a left ventricular lead (*Figure 1F*).

This is the first case report showing the feasibility of the balloon-target puncture technique for CRT upgrade in patients with SCV occlusion, departing from a similar approach for new pacemaker implantation.^1^ Before the CRT upgrade, 50% of patients may experience SCV occlusion related to the initial lead.^2^ Our balloon-supported method reduces the risk of damaging existing leads or vessel perforation while being less invasive than alternatives, such as tunnelling to the other side.

## Supplementary Material

ytae110_Supplementary_Data

## Data Availability

Data sharing is not applicable to this report because no datasets were generated or analysed for this case.
